# Low Salivary Testosterone Level Is Associated With Efficient Attention Holding by Self Face in Women

**DOI:** 10.3389/fnbeh.2019.00261

**Published:** 2019-11-29

**Authors:** Hirokazu Doi, Kazuyuki Shinohara

**Affiliations:** Graduate School of Biomedical Sciences, Nagasaki University, Nagasaki, Japan

**Keywords:** self, face, attention, testosterone, sex difference

## Abstract

Capacity to recognize one’s own face (hereinafter referred to as self face) is a fundamental component of various domains of social cognition such as empathy in humans. Previous research has demonstrated that a high level of androgen suppresses empathic behavior and social brain function. Taking these into consideration, we hypothesized that people with high androgen level show reduced response to self face. The present study examined this hypothesis by investigating the association between attentiveness towards self face, as assessed by a psychophysiological experiment, and salivary testosterone concentration. The attentional responses to self face was measured by a modified Go/NoGo task. In this task, self face or unfamiliar other’s face was presented simultaneously with Go or NoGo signal. In go trials, participants had to divert their attention from the face to a peripheral target. The reaction time (RT) for peripheral target detection in each condition was measured. In addition to behavioral data, saliva samples were collected to assay salivary testosterone concentration. The index of potency of self face to hold viewer’s attention that was computed based on RT data was regressed against salivary testosterone concentration in men and women separately. The analyses revealed that self face holds visuospatial attention more effectively in women with low than high salivary testosterone level, but no such trend was observed in men. This pattern of results indicates that low testosterone level is associated with a pronounced response to self face as we hypothesized and raises the possibility that multiple aspects of self-face processing are under the influence of endocrinological function.

## Introduction

Self-awareness is considered a cornerstone of social cognition (Gallup, 1970; Keenan et al., 2000; Humphreys and Sui, 2016). The distinction between self and other is indispensable in the theory of mind and perspective taking (Happé, 2003; Bradford et al., 2015). Reflecting this special status self holds in social cognition, one’s visual system processes self face in a manner different from an unfamiliar or a highly familiar other’s face. Tong and Nakayama (1999) demonstrated that the representation of self face is highly viewpoint invariant. In addition, many neuroimaging studies have revealed increased activation of neural regions, such as the medial prefrontal cortex and anterior cingulate cortex, to self faces compared with other’s faces (Keenan et al., 2000; Kircher et al., 2001; Heatherton et al., 2006), which indicates that exposure to self face induces introspection and emotional reaction effectively. Interestingly, part of these neural regions is recruited in inference of other’s mental status and perspective taking as well (Mitchell et al., 2005; Healey and Grossman, 2018), which gives further credence to the view that self-face processing comprises the basis of social cognition (Happé, 2003).

One phenomenon reflecting the special status of self face is its effectiveness to hold one’s attention (Humphreys and Sui, 2016). Psychophysical and eye-tracking studies have shown that self face holds adult’s attention more effectively than an unfamiliar or a familiar other’s face (Brédart et al., 2006; Devue et al., 2009; Humphreys and Sui, 2016; Wójcik et al., 2018).

There is great interindividual variation in the ability of social cognition. Such individual differences presumably stem from various factors, including environmental and biological factors. Among these, many studies point out that a high level of androgen is associated with poorer function in many domains of social cognition (van Wingen et al., 2011). Direct evidence for the link between social cognition and androgen comes from testosterone administration studies. This line of study has shown that a single administration of testosterone reduces empathy and mentalizing (Hermans et al., 2006; van Honk et al., 2011; but see Nadler et al., 2019). Correlational studies also linked a high level of testosterone to impaired social function, indicating the possibility that testosterone can impair social cognition ability even within the physiological range (Ronay and Carney, 2013; Zilioli et al., 2015).

Taken together, these pieces of evidence indicate that a high level of testosterone leads to a lower level of social cognition ability. Taking this into consideration, together with the proposed link between social cognition and self-processing (Happé, 2003), it is highly conceivable that a high level of testosterone is associated with reduced behavioral and neurophysiological responses to self-related information, including self face. However, the association between neuroendocrinological function and self-face processing has not been examined fully with only a few exceptions that investigated the association between representation of self face and levels of hormones (Colonnello et al., 2013; Welling et al., 2016). Specifically, Colonnello et al. (2013) revealed that intranasal administration of oxytocin increases one’s ability to discriminate self and other’s faces, while Welling et al. (2016) demonstrated that testosterone administration makes one’s representation of self face more masculine than the actual self face. Thus, despite the abundance of studies that indicate effectiveness of self face to hold viewer’s visuospatial attention (Devue et al., 2009; Humphreys and Sui, 2016; Wójcik et al., 2018), no study to date has investigated the link between the indicators of endocrinological function and attentional responses to self face.

The present study attempts to fill in the gap in knowledge by investigating the association between salivary testosterone concentration and strength of attention holding by self face to examine the hypothesis that a high level of testosterone is associated with weaker attention holding by self face. We collected data from men and women during their 20–30 s because many previous studies found link between testosterone level and sociocognitive function in population within similar age range (Welling et al., 2007, 2016; van Honk et al., 2011; Volman et al., 2011).

## Materials and Methods

### Participants

The present study included 44 males (mean age = 20.7 years old, *SD* = 2.9; age range = 18–32) and 36 females (mean age = 21.3 years old, *SD* = 3.3; age range = 18–35) participants with normal or corrected to normal visual acuity after they gave written informed consent. Most of them were in their early 20 s. There was no significant between-group difference in age, *t*_(78)_ = 0.82, *p* = 0.42, *d* = 0.18. Participants with history of psychiatric and neurological conditions or being currently on medication were excluded from the final sample.

### Procedure

#### Behavioral Experiment

After the participants arrived at the lab, we took a picture of each participant’s face against cream-white background. The participants were instructed to maintain a neutral expression with their mouths closed. The image was cropped and adjusted in size so that the resultant image fit an 8.6 cm × 8.6 cm square that served as the face stimulus in the Self condition. Face stimuli presented in the Other condition were created by averaging 30 faces of people with roughly the same age (20–30 s) as the participants. The unfamiliar face for female participants was created from 30 female faces while that for male participants was created from 30 male faces. The identical same-sex average face was presented for all the participants in each sex in the Other condition because the participants’ age was not widely distributed. The size of the face image in the Other condition was equal to that in the Self condition. The stimuli were presented on a 17-inch monitor viewed from ~65 cm away.

**Figure 1:**
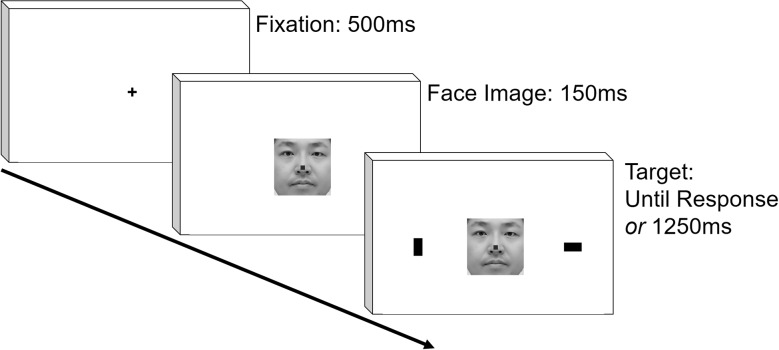
Schematic representation of temporal sequence in stimulus presentation. The face image is not exactly the same with those presented in the actual experiment.

After instructions were given to the participants, the experiment started. At the start of each trial, a fixation cross appeared at the center against white background for 500 ms. Then, a face image was presented. In two-thirds of the trials, a small green square subtending 0.67 cm was presented at the height of the nose (Go condition), and a small red rectangle was presented in the remaining trials (NoGo condition). One-hundred and fifty milliseconds after the presentation of the face image, two 1.3 cm × 2.3 cm black rectangles were presented at the periphery of screen, ~11.8 cm from the center, simultaneously with the face image. One was presented in vertical, and the other in horizontal orientation. In the Go condition, the participants identified the location of the horizontal target using a key press as soon as possible. In the NoGo condition, they were instructed to refrain from making any responses. The targets stayed on the screen for 1,250 ms in the NoGo trials. We included Go and NoGo conditions so that the participants would pay attention to the face image; we wanted to make sure that participants direct overt attention to the facial image because there was a good chance that they might process the faces only in parafoveal vision due to relatively large size of the facial images used. After the key press or 1,250 ms passes after the appearance of face image, feedback about the correctness of response was presented for 300 ms. There was no intertrial interval. Thus, after the disappearance of feedback, the experiment immediately proceeded to the next trial. The sequence of the stimulus presentation is schematically shown in Figure 1. There were 32 Go and 16 NoGo trials each for the Self and Other conditions, yielding in total of 96 trials. The trials in these conditions were pseudorandomly ordered with the restriction that trials of the four conditions (Go/NoGo × Self/Other) were evenly distributed throughout the experiment.

#### Salivary Sample Collection

The saliva sample was collected between 12:00 and 14:00 h to mitigate the influences of circadian fluctuation (Dabbs, 1990). Each saliva sample was deposited into a polystyrene tube by passive drool and stored at −80°C until the assay. The participants refrained from eating, drinking, smoking, brushing their teeth, and exercising for 1 h before the experiment. They also rinsed their mouths with water ~15 min before the sample collection.

#### Self-administered Questionnaires

After the behavioral experiment, participants were asked to complete the Japanese translation of Rosenberg’s self-esteem scale (Rosenberg, 1965) and the self-consciousness scale (Sugawara, 1984). We collected data of these questionnaires because attitude to and one’s evaluation of self might influence attentional responses to self face. Self-esteem scale is comprised of ten 5-point items that measure the level of positive evaluation of one’s worth and abilities (range = 5−50; Yamamoto et al., 1982). Self-consciousness scale includes 7-point items that measure private (range = 7−70) and public self-consciousness (range = 7–77; 10 items for private self-consciousness and 11 items for public self-consciousness). Private self-consciousness refers to the tendency to pay attention to own inner states, while public self-consciousness is the tendency to pay attention to own appearance and how one’s behavior is evaluated by others. The self-consciousness scale is a modified version of Feininger’s inventory (Fenigstein et al., 1975) but includes items more familiar to the Japanese population. Still, it shows a reliable two-dimensional structure of public and private self-consciousness (Fenigstein et al., 1975).

### Testosterone Concentration Analysis

After all participants had completed the experimental tasks, the concentration of salivary testosterone in each sample was assayed by enzyme immunoassay (EIA) using a commercially available kit (Salimetrics Europe Limited, Suffolk, UK). Testosterone level in saliva samples is known to correlate with serum testosterone level in men but not necessarily in women. At the same time, salivary concentration of testosterone is supposed to reflect the level of free-testosterone and testosterone only weakly binding to sex hormone-binding globulin and hence is considered to be a reliable indicator of the level of bioactive testosterone (Papacosta and Nassis, 2011). The sample was first centrifuged and the aqueous layer was aliquoted for assay. Information about the recovery and specificity of the kit can be found online in the EIA kit manual. In short, testosterone concentration in 25 μl of undiluted saliva samples was measured by competitive immunoassay. The optical densities of each well of the plate was read by a microplate reader at 450 nm and then converted to testosterone concentration values on the basis of simultaneously measured standard curve. The percent cross-reactivity with estradiol, progesterone, and cortisol is reported to range from ND (none detected) to <0.03 (see for more details, https://salimetrics.com/wp-content/uploads/2018/03/testosterone-saliva-elisa-kit.pdf).

### Statistical Analysis

The potency of self face to hold a participant’s visuospatial attention was quantified as the standardized difference between reaction time (RT) in Self-Go and Other-Go conditions using the following equation:

$${\text{RT}}_{\text{diff}}=\frac{{\text{RT}}_{\text{self}}-{\text{RT}}_{\text{other}}}{{\text{RT}}_{\text{self}}+{\text{RT}}_{\text{other}}}$$

where RT_self_ and RT_other_ are mean RT in successful trials in Self-Go and Other-Go conditions, respectively. Higher RT_diff_ indicates less efficient disengagement of visuospatial attention from self than other’s face, thus more efficient holding of attention by self face. We obtained essentially the same results when analyzing RT_self_ − RT_other_ without standardization. Thus, in the following, we report only the results of RT_diff_.

We carried out linear and quadratic regression analyses separately for male and female participants because previous studies found sex differences in androgenic effects on higher-order cognition (Moffat and Hampson, 1996; Sapienza et al., 2009; Doi et al., 2015, 2018). We also carried out two-way between-participant analysis of variance (ANOVA) and *t*-tests for group comparisons. All the statistical analyses were carried out using R 3.5.2 (R Development Core Team). The power was computed by G*Power 3.1 (Faul et al., 2007) using medium effect size (Cohen, 1988).

## Results

### Sex Difference

We first examined sex differences in hormonal and behavioral measures. The mean and standard deviations of these variables are summarized in Table 1. The range of salivary testosterone concentration was comparable to the previous studies (Deady et al., 2006; Welling et al., 2007; Cobey et al., 2015). As expected, the salivary testosterone concentration was significantly higher in male than in female participants, *t*_(78)_ = −16.2, *p* < 0.001, *d* = 3.79. No other comparison reached statistical significance, *t*s < 1.54, *p*s > 0.12.

**Table 1:** The means and standard deviations of hormonal and behavioral results.

	Testosterone (pg/ml)	RT_Self_ (ms)	RT_Other_ (ms)	Self-Esteem	Public	Private
Male	262.7** (65.3)	533.4 (76.7)	535.9 (81.4)	31.8 (7.6)	52.6 (10.2)	46.0 (8.5)
Female	77.2 (22.7)	562.4 (92.0)	562.4 (101.7)	29.6 (6.7)	55 (10.8)	46.4 (8.5)

The standard deviations are in the parenthesis. RT_Self_, reaction time in Self-Go condition; RT_Other_, reaction time in other-Go condition; Public, public self-consciousness; Private, private self-consciousness, **p < 0.01 in group comparison.

### Association Between Testosterone and Attentiveness Toward Self Face

**Figure 2:**
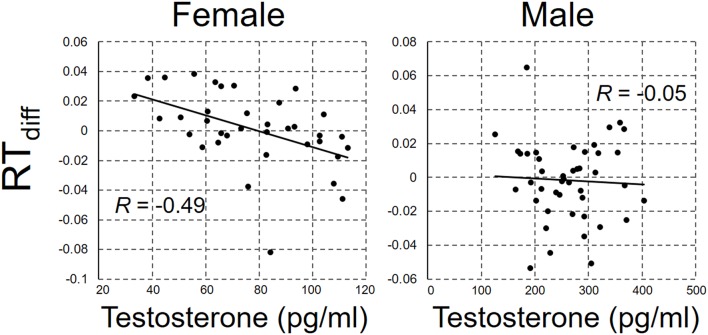
The scatterplot between RT_diff_ and salivary testosterone concentration for female and male participant.

RT_diff_ was regressed against the salivary testosterone concentration. The scatterplots between RT_diff_ and salivary testosterone concentration are shown in Figure 2 for male and female participants separately. There was a significant negative correlation between RT_diff_ and testosterone concentration in female participants, *r*_(34)_ = −0.49, *p* = 0.003, but not in male participants, *r*_(42)_ = −0.05, *p* = 0.76, power = 0.71.

To clarify the nature of this pattern of correlational analysis, three additional analyses were conducted. In the first analysis, we carried out multiple regression analysis for data of female participants with RT_diff_ as the dependent variable. The predictors included testosterone concentration, age, and questionnaire results of self-esteem, public self-consciousness, and private self-consciousness. The results are summarized in Table 2. As can be seen in the table, the correlation between testosterone concentration and RT_diff_ persisted even after the influences of the other predictors were controlled for.

**Table 2:** Summary of the statistical values of the multiple regression analysis.

	*β*	SE	*t*-value	*p*-value
Testosterone	−0.512	0.167	−3.072	0.005
Age	0.141	0.175	0.808	0.426
Self Esteem	−0.185	0.186	−0.994	0.329
Public	−0.201	0.183	−1.102	0.28
Private	−0.029	0.184	−0.158	0.876
Intercept	−0.012	0.163	−0.076	0.94

*β, standardized coefficient of each predictor; SE, standard error of each predictor; Public: public self-consciousness; Private, private self-consciousness*.

From the visual inspection of scatterplot (Figure 2), there seems to be a curvilinear trend in the relationship between testosterone concentration and RT_diff_ in male participants. Considering this together with the previous study indicating curvilinear relationship between androgen and behavior (Moffat and Hampson, 1996; Tan and Tan, 1998; Sapienza et al., 2009; Doi et al., 2015; for a review see Swift-Gallant and Monks, 2017), in the second analysis, we carried out a quadratic regression analysis with RT_diff_ as the dependent variable and testosterone concentration and squared testosterone concentration as the independent variables. The quadratic model did not show significant fit to RT_diff_, *F*_(2,41)_ = 1.47, *p* = 0.24, *r*^2^ = 0.07, power = 0.59.

**Figure 3:**
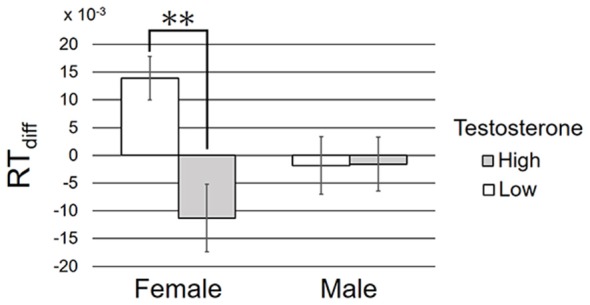
The mean and standard deviation of RT_diff_ in each condition. **p < 0.01 in the simple main effect analysis.

In the third analysis, participants were first classified into high and low testosterone groups within each sex. The median value of testosterone concentration within each sex was used as the criteria of participant grouping. For example, female participants whose testosterone level was higher than the median testosterone concentration in all the female participants were included into high-female group. Then, we submitted RT_diff_ to a two-way between-participant ANOVA with factors of sex (2) and testosterone (2; high–low). The mean and standard deviation in each group are shown in Figure 3.

ANOVA revealed a significant main effect of testosterone, *F*_(1,76)_ = 5.97, *p* = 0.017, $\eta_{\text{p}}^{2}$ = 0.073, but the main effect of sex failed to reach significance, *F*_(1,76)_ = 0.35, *p* = 0.56, $\eta_{\text{p}}^{2}$ = 0.004. These effects were qualified by a significant interaction between sex and testosterone, *F*_(1,76)_ = 6.21, *p* = 0.015, $\eta_{\text{p}}^{2}$ = 0.076. Simple main effect analysis revealed RT_diff_ as significantly higher in female participants with low rather than high salivary testosterone concentration, *F*_(1,76)_ = 11.07, *p* = 0.0014, $\eta_{\text{p}}^{2}$ = 0.13. No such effect was found in male participants, *F*_(1,76)_ = 0.001, *p* = 0.97, $\eta_{\text{p}}^{2}$ < 0.001, power = 0.38. For explanatory purpose, we tested whether the averaged RT_diff_ differed from zero. *T*-tests revealed significant deviation of RT_diff_ from zero in female participants with low testosterone, *t*_(17)_ = 3.55, *p* = 0.002, but not in the other three groups, *t*s < 1.9, *p*s > 0.08, powers > 0.51.

## Discussion

Many studies have shown the association between androgenic function and cognitive/perceptual abilities such as spatial perception, financial decision making, and aggression (Moffat and Hampson, 1996; Mazur and Booth, 1998; Sapienza et al., 2009; Doi et al., 2015). The ability of social cognition is no exception to this, and an accumulating number of studies has linked a higher level of testosterone with poorer ability in many domains of social cognition (Welling et al., 2016; van Honk et al., 2011; Ronay and Carney, 2013; Zilioli et al., 2015; but see Nadler et al., 2019). Given the close linkage between processing of self-related information and sociocognitive functions (Happé, 2003; Bradford et al., 2015), it seems plausible to postulate an association between testosterone level and self-related information processing.

The present study revealed that female participants with low salivary testosterone show inefficient disengagement of attention from self face compared with those with a relatively high testosterone level. In other words, self face holds attention more efficiently in female participant with low than high testosterone level. Actually, female participants with low testosterone level was the only group that showed self-face advantage in attention holding in the present study in line with the previous findings (Devue et al., 2009; Humphreys and Sui, 2016; Wójcik et al., 2018). Furthermore, efficiency of attentional disengagement from self face was not related to any variables tested other than testosterone level in female participants. Taken together, these observations seemingly indicate that a high testosterone level is associated with a reduced response to self face as hypothesized. Although many studies have revealed androgenic influences on social cognition, to the best of our knowledge, this is the first to empirically show the relationship between systemic androgen levels and the attentional response to self face.

Some neural regions recruited in self-face processing, such as the medial prefrontal cortex and amygdala, are rich with androgen receptors in mammals (Simerly et al., 1990; Finley and Kritzer, 1999), and a previous study has found functional decoupling of these regions by testosterone administration (Volman et al., 2011). Thus, downregulation of functional connectivity in this neural network probably explains the reduced attentiveness toward self face in the present study. A previous study showed that men administered with exogenous testosterone tend to have an inner representation of their own face that is more morphologically masculine than what is actually true (Welling et al., 2016). Taken together with this, the present study indicates that androgen modifies multiple aspects of self-face processing.

Together with the previous findings associating high level of testosterone with impaired socio-cognitive abilities (Hermans et al., 2006; van Honk et al., 2011; Ronay and Carney, 2013), the overall pattern of the present results seemingly supports the proposed link between self-face processing and social cognition (Happé, 2003). However, we did not collect measures of the other aspects of social cognition such as empathy and perspective taking because the primary aim of the present study was not to validate the link between self-face processing and social cognition in general. As a result, it remains unclear whether testosterone affects all aspects of social cognition including self-face processing in the same way. With regard to this point, the extreme male brain theory of autism (Baron-Cohen, 2010) claims that hypermasculinization of brain induced by exposure to excessive level of androgen shower during fetal period leads to later impairment in empathic behavior while promoting systemizing tendency. Furthermore, many researchers argue that high level of fetal androgen exposure decreases second/fourth digit length ratio (2D:4D; Manning et al., 1998; Hönekopp et al., 2007). On the basis of these, if self-face processing is intrinsically linked to social cognition, stronger attention holding by self face should be observed in people with high compared to low 2D:4D. Thus, multiple measures of social cognition and 2D:4D should be incorporated to get a more comprehensive picture of the relationship between self-face processing, social cognition, and androgen. In relation to this, it would also be of interest to see if individual difference in self-face processing is related to the empathizing–systemizing cognitive styles (Baron-Cohen, 2009).

Interestingly, we found no robust relationship between behavioral performance and salivary testosterone concentration in men. RTs in women were numerically longer than men irrespective of conditions. Short RT in men may have resulted in a kind of floor effect that masked any association between testosterone level and behavioral effect. A sex difference in sensitivity to the activational effect of androgen has often been reported in previous studies (Moffat and Hampson, 1996; Sapienza et al., 2009; Doi et al., 2015, 2018), but its cause remains largely unknown. One possible explanation is that self-face processing of male young adult, whose brain had already been masculinized/defeminized to some extent during the fetal period (Baron-Cohen, 2010), is not modified further by subtle difference in the level of circulating testosterone within physiological range. The social brain is saturated with androgens in this population at this stage of life, so differences in endogenous testosterone level may not have observable effect on self-related information processing. However, at this point, this is mere speculation and should be validated with empirical results. An alternative explanation is the often-reported curvilinearity or plateauing due to ceiling effect in the relationship between androgen and behavior (Swift-Gallant and Monks, 2017). This explanation is partly refuted in the present dataset because a quadratic regression model including squared testosterone concentration as the predictor failed to show correlation with self-face advantage (RT_diff_). However, this may be because of relatively weak power of statistics in the present study.

In contrast to women with low testosterone, those with high testosterone level showed tendency to be more attentive to unfamiliar other’s face than self face. A previous study found that testosterone administration reduces perceived trustworthiness of others’ faces in women (Bos et al., 2010). Given that threatening images are the most potent stimuli to capture attention (Mogg and Bradley, 2010), the observed tendency in women with high testosterone seemingly stems from reduced trust in unfamiliar others.

Lack of clear self-face advantage in men was totally unexpected. There are several explanations for this null result. First, our previous study (Doi and Shinohara, 2018) has shown that male young Japanese do not show clear attentional prioritization of self face over other’s face after mid-adolescence. Taking into consideration the previous finding indicating that developmental change of face processing continues into late adulthood (Anastasi and Rhodes, 2005; Boutet et al., 2015), we might get a different picture if, we recruit younger or older population. The second potential cause is that the stimulus onset asynchrony (SOA) between face image and target stimuli was not optimal to detect self-face advantage in attentional responses in men. Previous studies on the influences of facial information on visuospatial attention have revealed that the effect of facial information on behavioral response is sensitive to SOA (Liu et al., 2016; Carlson et al., 2019). Thus, it is necessary to test the association between testosterone and attentional responses to self face using more varying SOAs in the future study.

We presented averaged face of same-sex persons as unfamiliar other’s face. Average faces are generally perceived to be attractive (Little et al., 2011) From the perspective of evolutionary psychology, attractive face signals health and high reproductivity (Rhodes, 2006). Considering this, it is possible that participants have implicitly deemed averaged same-sex face as potential competitor for resources and sexual mates (Ellis, 2006). Such intrasex competition might have increased attentiveness to other’s face especially in men, who are reported to show stronger tendency of intrasexual competition (Ellis, 2006), which might explain the lack of self face advantage in men. Several studies have shown that the attributes (age, sex) of viewers interact with those of viewed faces in determining the pattern of neural and behavioral responses to other’s face (Doi et al., 2010; Hills and Lewis, 2011; Kret and De Gelder, 2012; Rhodes and Anastasi, 2012). For example, Doi et al. (2010) revealed that the amplitude of an event-related potential component reflective of emotional and attentional responses to face increases in response to the same-sex compared to opposite-sex faces with neutral expression. In the present study, we used average faces of same-sex people within age range similar to participants to match the attributes of self and other’s face closely. However, we cannot deny the possibility that this specific choice introduced some complications to the results in the present study.

There are several limitations that qualify the interpretation of this study. First, this is a mere correlational study, and thus, we cannot ascertain the causal linkage or direction between testosterone and attentiveness to self face. To establish the presumed causal linkage, a testosterone administration study on attentiveness to self face is warranted. Second, we did not collect information on menstrual cycle in female samples. Testosterone can exert influences on neural function through aromatization into estrogen (Roselli et al., 2010). Thus, the effect of testosterone might be confounded by fluctuations in secretion levels of other hormones such as estrogen and progesterone, as might be the levels of testosterone itself. These hormones could confound the results for men as well. Furthermore, the level of testosterone itself fluctuates during menstrual cycle. Thus, simultaneous measurement of multiple hormones should be required in the future study to see whether the attentional response to self face is specifically linked to testosterone level or not.

## Conclusion

In summary, the present study investigated the association between attentiveness to self face and salivary testosterone concentration. The results revealed that self face holds visuospatial attention of female individuals with low testosterone level more effectively than those with high testosterone concentration. This finding gives support to the view that self-face processing, a fundamental component of social cognition, is also under the influences of androgenic function.

## Data Availability Statement

The datasets generated for this study are available on request to the corresponding author.

## Ethics Statement

The studies involving human participants were reviewed and approved by the Ethics Committee of Graduate School of Biomedical Sciences, Nagasaki University. The patients/participants provided their written informed consent to participate in this study. Written informed consent was obtained from the individual(s) for the publication of any potentially identifiable images or data included in this article.

## Author Contributions

HD conceived the study, carried out data collection and analysis, and wrote the manuscript. KS assisted in data collection and hormone assay and approved the manuscript.

## Conflict of Interest

The authors declare that the research was conducted in the absence of any commercial or financial relationships that could be construed as a potential conflict of interest.
